# Molecular Simulation Study on the Impact of a Cross-Linked Network Structure on the Tensile Mechanical Properties of PBT Substrates

**DOI:** 10.3390/ma18071675

**Published:** 2025-04-06

**Authors:** Renlong Huang, Kang Zhao, Peng Cao, Liang Cao, Hongjun Liao, Xianqiong Tang

**Affiliations:** 1 School of Mechanical Engineering and Mechanics, Xiangtan University, Xiangtan 411105, China; 202221572230@smail.xtu.edu.cn (R.H.); 202221572238@smail.xtu.edu.cn (H.L.); 2 The Institute of Xi’an Aerospace Solid Propulsion Technology, Xi’an 710025, China; kangzhao@stu.xjtu.edu.cn; 3 College of Architecture and Civil Engineering, Beijing University of Technology, Beijing 100124, China; caopeng518888@126.com (P.C.); liangcao988@126.com (L.C.)

**Keywords:** molecular dynamics, cross-linked lattice, mechanical properties, uniaxial stretching

## Abstract

This study investigates the correlation between the cross-linked network structure and the macroscopic mechanical properties of 3,3-bis(azidomethyl)oxetane-tetrahydrofuran copolymer (PBT)-based solid propellants through molecular dynamics (MD) simulations. A multi-component system comprising PBT molecular chains, toluene diisocyanate (TDI), trimethylolpropane (TMP), tetraethylene glycol (TEG), and sodium perchlorate (AP) was constructed. Perl script programming was utilized to precisely control the dynamic cross-linking reaction. Molecular models with cross-linking densities of 0%, 50%, 60%, 70%, 80%, and 90% were established, and their mechanical properties were analyzed under varying cross-link densities and strain rates through uniaxial tensile simulations. The results indicate that the formation of the cross-linked network significantly alters the energy distribution and microstructural characteristics of the system. As the cross-linking density increases from 50% to 90%, the total energy of the system decreases by approximately 40%, primarily due to reductions in non-bonded energy. The radial distribution function (RDF) and root mean square displacement (MSD) curves reveal that the cross-linking reaction enhances covalent bond formation between molecular chains, reduces their degrees of freedom, and increases the glass transition temperature (*Tg*). Under identical strain conditions, the models with higher cross-link densities exhibit greater stress resistance. Specifically, the stress growth rate of the 90% cross-link density system increases by 42.1% as the stretching rate rises from 1.0 × 10^11^ s^−1^ to 2.0 × 10^11^ s^−1^, compared to an 18.7% increase for the 50% cross-link density system. These findings have significant implications for optimizing processing parameters and predicting the mechanical properties of propellants.

## 1. Introduction

**Figure 1:**
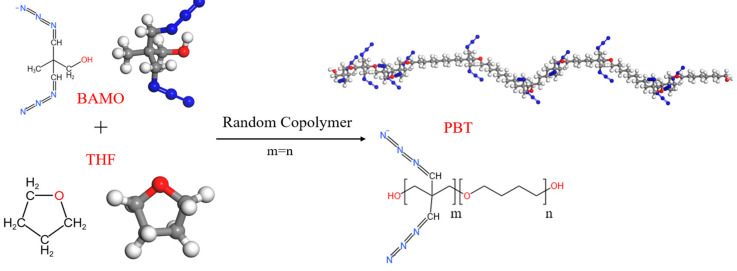
Schematic diagram of PBT synthesis and molecular structure. Color scheme: Gray (Carbon), Blue (Nitrogen), Red (Oxygen), White (Hydrogen).

Azide polyether propellant represents a novel high-energy solid propellant system that has emerged as a leading area of research in solid propellant technology. This is attributed to its numerous advantages, including a low characteristic signal, high specific impulse, low vulnerability, and adjustable energy content [1,2,3]. Among these, the azide-poly(3,3-bis(azidomethyl)oxetane-co-tetrahydrofuran) (Poly(BAMO-co-THF) (abbreviated as PBT)-based propellant has gained significant traction in engineering applications due to its exceptional overall performance. The polymer matrix is synthesized through the copolymerization of the high-energy-density monomer 3,3-bis(azidomethyl)oxetane (BAMO) with tetrahydrofuran (THF), resulting in a material with a low glass transition temperature. The flexible–rigid synergy of the molecular chains endows the propellant with superior mechanical properties at low temperatures [4,5]. The synthetic components and molecular structure of PBT are shown in Figure 1.

To enhance the mechanical properties of the PBT matrix, researchers commonly employ trimethylolpropane (TMP) as a cross-linking agent, 2,4-toluene diisocyanate (TDI) as a curing agent, and triethylene glycol (TEG) as a chain extender. By varying the isocyanate index, a three-dimensional network structure with an adjustable cross-linking density is formed [6]. TDI, as an isocyanate curing agent, facilitates the linkage of linear PBT molecular chains into a three-dimensional network through an additional reaction with hydroxyl groups [7]. TEG extends the molecular chains by reacting with isocyanate groups, thereby minimizing defects caused by incomplete reactions or unreacted functional groups, which results in a more uniform and stable polymer structure [8]. TMP, as a trifunctional cross-linking agent, enhances the formation of the cross-linked network in systems such as PBT/TDI. Its trifunctional nature allows TMP to act as a branching point, connecting multiple polymer chains and reinforcing the overall material structure [9]. Additionally, to ensure high energy efficiency in practical applications, an oxidant is incorporated. Ammonium perchlorate (AP), a typical solid oxidant, not only provides oxidation capacity but also serves to fill and plasticize the matrix [10]. The incorporation of AP into the PBT-TDI-TMP-TEG cross-linked network improves interfacial adhesion and promotes load transfer within the composite, thereby enhancing the mechanical properties of the matrix [11,12].

Currently, research on the structural–property relationships of PBT-based propellants predominantly integrates experimental characterization with theoretical modeling. Formulation systems with varying BAMO/THF molar ratios, TMP contents, and DEG/TDI ratios are designed, and their mechanical properties and microphase separation behaviors are quantitatively characterized using techniques such as uniaxial tensile tests, dynamic mechanical analysis (DMA), and differential scanning calorimetry (DSC) [13,14]. Aleksandra [15] utilized Fourier transform infrared spectroscopy (FTIR) to analyze the degree of hydrogen bonding and combined this with wide-angle X-ray diffraction (WAXD) to investigate changes in crystallinity. This study revealed the influence of different diisocyanate types (such as MDI, TDI, IPDI, and HDI) on the reconstruction of the hydrogen bond network and the micro-morphology of polyurethane adhesives. Wang [16] demonstrated that the type of diisocyanate affects the crystallinity and micro-morphology of polyurethane adhesives, which subsequently influences their mechanical and thermal properties. However, the existing experimental and modeling approaches often struggle to elucidate the intrinsic correlations between the topological parameters of the cross-linking network (such as the spatial distribution of cross-linking points, segment entanglement density, and dynamic evolution) and the macroscopic mechanical response at the molecular scale. This challenge is particularly pronounced in complex multi-component systems like PBT-based propellants, where traditional experimental methods are limited by resolution and cannot directly observe energy changes at active sites during cross-linking reactions, the formation of network defects, and the cross-scale evolution of stress transmission paths. Furthermore, the existing theoretical models frequently assume a continuous medium and fail to fully account for nanoscale effects, such as changes in the conformational entropy of molecular chains and the dynamic dissociation and association of hydrogen bonds, which contribute to nonlinear viscoelastic behavior [17,18,19].

In recent years, the rapid advancement of molecular simulation technology has opened new avenues for analyzing the multi-scale structure–property relationships of polymer networks. By constructing accurate coarse-grained or full-atom force fields, molecular dynamics (MD) simulations can quantitatively track functional group reaction pathways, cross-linking density gradient distributions, and molecular chain orientation evolution during the formation of cross-linked networks [20,21]. For instance, Shundo et al. [22] employed MD simulations to explore the regulatory effect of local cross-linking density heterogeneity on the glass transition temperature during the curing process of an epoxy resin system. Bugrov et al. [23] confirmed through a coarse-grained model that the size and distribution of the hard segments in a polyurethane network influence its mechanical properties, with these hard segments acting as physical cross-links that enhance the material’s stiffness and elastic behavior. Zhao et al. [24] utilized density functional theory (DFT) to investigate the curing mechanism of PBT and TDI from the perspectives of the molecular structure, electronic properties, kinetic parameters, and potential energy surfaces. Dong [25] studied the interfacial interactions between ammonium perchlorate (AP) and a hydroxyl-terminated polybutadiene (HTPB) matrix through MD simulations, finding that the micro-cross-linking structure, stretching rate, and contact area between the filler and matrix significantly affect the mechanical properties of the composite solid propellant.

Despite these advancements, a systematic study of the dynamic construction mechanism of the cross-linking network in azide polyether propellants, particularly in PBT matrices, remains lacking. Specifically, the influence of the cross-linking density on the network stability, mechanical properties, energy changes during the cross-linking process, and glass transition temperature is still not well understood. Therefore, this study employs MD simulations to construct PBT matrix networks with varying cross-linking densities and investigates the mesh structure and mechanical properties at different tensile rates. This work is organized into four sections. Section 2 details the establishment of the PBT azide cross-linked body, ammonium perchlorate (AP) crystal, cross-linking model, and conditions for the uniaxial tensile simulations. Section 3 provides a comprehensive analysis of the energy, radial distribution function (RDF), diffusion coefficient, and glass transition temperature of the structure across different cross-linking densities. Additionally, the stress–strain curves corresponding to varying cross-linking densities and tensile rates are discussed in detail. Finally, Section 4 concludes with a summary of the findings from this study.

## 2. Model Construction and Simulation Details

The simulation was conducted using the molecular dynamics software Materials Studio 2020 (MS 2020). The COMPASS II force field [26,27,28] was selected for the simulation. This quantum mechanics-based force field is capable of accurately predicting the structural, conformational, vibrational, and thermophysical properties of a broad range of monomolecular and polymerized systems. It is applicable to both organic and inorganic materials, including polymers and heterocyclic compounds. The simulation includes three main steps. First, molecular models are constructed for a specific proportion of the PBT molecular chain, curing agent TDI, cross-linking agent TMP, chain extender TEG, and oxidant AP. Next, the dynamic cross-linking reaction is simulated through Perl script programming, enabling the automatic cross-linking of the aforementioned components and the generation of a three-dimensional mesh structure. The model is then geometrically optimized using the Smart algorithm with a convergence tolerance of 0.001 kcal/mol (energy), 0.005 kcal/mol/Å (force), and 0.005 Å (displacement) and a maximum of 10,000 iterations to ensure convergence. Finally, performance prediction is performed, starting with the high-temperature annealing of the cross-linked molecular model. This is followed by simulations involving different stretching rates to analyze the structural and mechanical properties.

### 2.1. Cross-Linking Modeling

**Figure 2:**
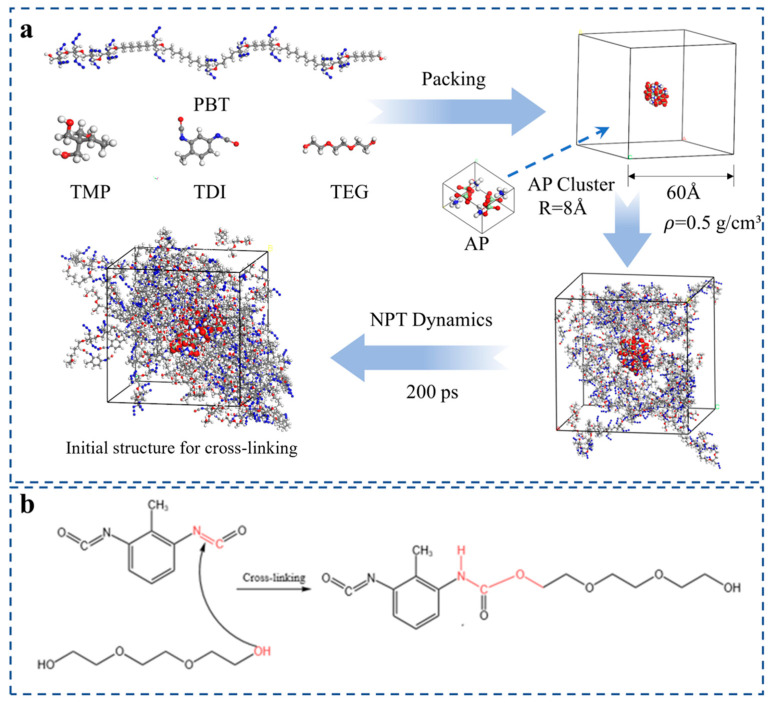
(**a**) Basic molecular model diagram and initial model construction diagram before cross- linking; (**b**) schematic diagram of cross-linking reaction. Color scheme: Gray (Carbon), Blue (Nitrogen), Red (Oxygen), Gyan (Chlorine), White (Hydrogen).

**Figure 3:**
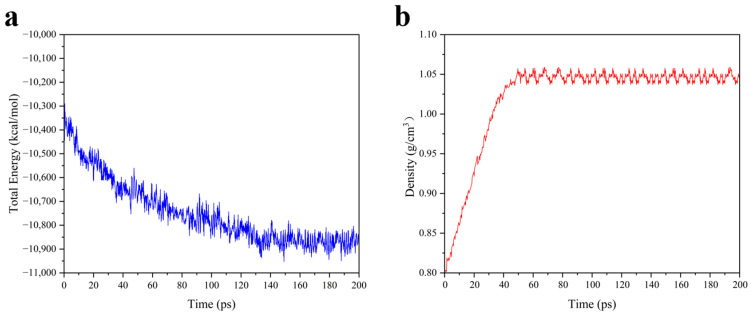
The 50% cross-linking density NPT molecular dynamics simulation process. (**a**) Energy–time curve; (**b**) density-time curve.

As illustrated in Figure 2a, after constructing the PBT long-chain polymer, the components were placed into a cubic simulation box containing ammonium perchlorate (AP) clusters. The components were distributed according to the molar ratio of the active functional groups using the packing function of the Amorphous Cell module, with an initial density set to 0.8 g/cm^3^. Prior to simulating the cross-linking reaction, global optimization was performed to eliminate high-energy regions. Subsequently, the molecular dynamics simulations were conducted using the COMPASS II force field in an isothermal–isobaric (NPT) ensemble, with a simulation duration of 200 ps and an integration step of 1 fs. Take the NPT dynamic simulation process with a 50% cross-linking density model as an example. During the simulation, the energy fluctuates with time until dynamic stability is reached, as shown in Figure 3a. As shown in Figure 3b, the density increases with time, reaches about 1.05 g/cm^3^, and remains dynamically fluctuating. The density value is similar to the PBT density synthesized from the literature experiments [29]. This proves that the model is reasonably constructed. The lowest-energy structure was obtained through optimization, resulting in a system density of 1.01 g/cm^3^. The final configuration comprised 14 PBT molecular chains, 42 TDI molecules, 28 TEG molecules, and 20 TMP molecules. This setup served as the initial configuration for subsequent cross-linking simulations. The PBT chains were designed to closely approximate the actual system based on the long-chain molecular properties of the prepolymer used in the experimental synthesis process. Specifically, a PBT chain structure consisting of 20 repeating units was selected, with a BAMO to THF ratio of 1:1, as detailed in [30].

Taking the isocyanate [-NCO] functional group of TDI and the hydroxyl [-OH] group of TEG as an example, the cross-linking reaction mechanism is illustrated in Figure 2b, with the main atoms and bonds involved in the reaction highlighted in red. The condensation reaction between the [-NCO] functional group of the TDI molecule and the terminal [-OH] group of the monomer proceeds as follows: the carbon atom (C) of the [-NCO] group is designated as the active site R1, while the oxygen atom (O) of the [-OH] group is designated as the active site R2. Step 1: Set the threshold interval to (4 Å, 5 Å). The Perl script calculates the distance between R1 and R2, and if the distance is within the threshold interval, change the C=O double bond in [-NCO] to a single bond, and a new bond will be created between R1 and R2. Step 2: set the threshold interval to (4 Å, 6 Å) and repeat step 1. Step 3: set the threshold interval to (4 Å, 7 Å) and repeat step 1. Step 4: set the threshold interval to (4 Å, 8 Å) and repeat step 1. Step 5: set the threshold interval to (4 Å, 9 Å) and repeat step 1. When the distance between the active sites falls below the critical threshold of 4 Å, molecular object tracking is halted to prevent intramolecular cross-linking and repetitive cross-linking. Conversely, when the distance exceeds 9 Å, no reaction occurs. A distance probability dual-control bonding algorithm was employed to regulate the reaction pathway.

**Figure 4:**
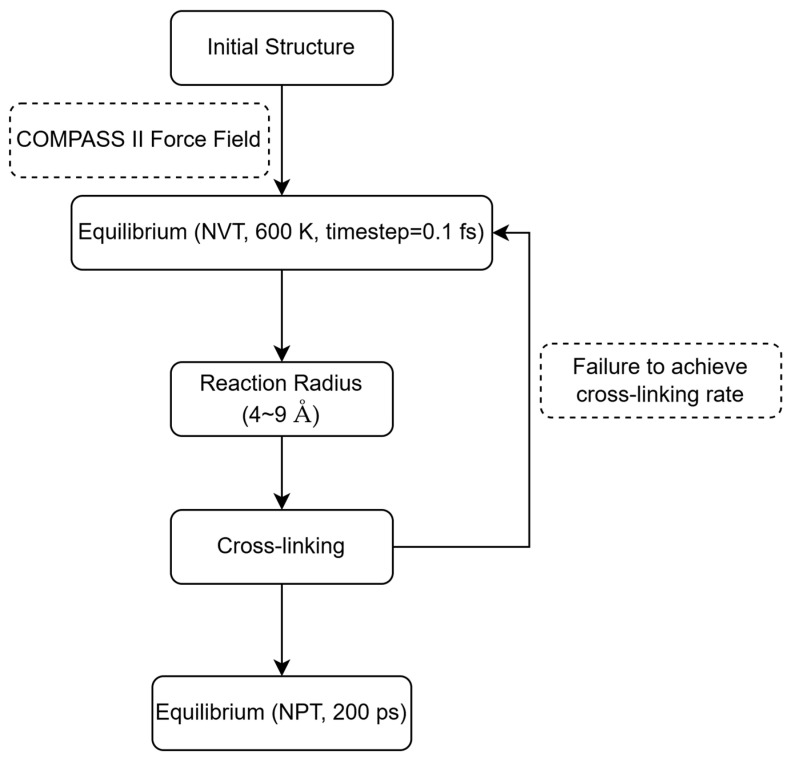
Initial model formation process for uniaxial tensile simulation.

To enhance the accuracy of the reaction process, the time step during the cross-linking phase was reduced to 0.1 fs. To ensure a complete reaction between the [-NCO] and [-OH] groups, the ratio of R1:R2 was set to 1:1.2. Bonding state detection was performed every 10 steps (1 fs) after the onset of cross-linking, with the reaction considered complete once the protons (H) from the [-OH] group migrated to the nitrogen atom (N) of the [-NCO] group following successful bonding. Five systems with varying cross-linking densities were constructed, corresponding to the cross-linking densities of 50%, 60%, 70%, 80%, and 90%, respectively. These systems were equilibrated at 300 K in an NPT ensemble for 200 ps, and the equilibrated models were subsequently used for tensile simulations. The process of constructing the cross-linking models is depicted in Figure 4.

### 2.2. Uniaxial Tensile Simulation

The uniaxial tensile simulation was conducted in two phases. The first phase involved applying uniaxial stretching along the *Z*-axis to each of the five models. The strain was incremented by 1% of the model’s side length with each application, continuing until the total strain reached 60%. During this complete cycle, a total of 2000 frames of data were recorded for each strain increment. The second phase focused on varying the strain rate. Strain rates of 1.0 × 10^11^, 1.2 × 10^11^, 1.4 × 10^11^, 1.6 × 10^11^, 1.8 × 10^11^, and 2.0 × 10^11^ s^−1^ were applied, with a total stretching duration of 300 ps for each rate. The calculations were performed using the Forcite module, with the NPT ensemble selected to maintain a constant temperature throughout the simulation.

An initial equilibrium run of 10,000 steps was conducted to eliminate the effects of the initial configuration and ensure that the system reached thermodynamic equilibrium. The trajectory saving frequency was set to every 2000 steps. During the formal stretching process, kinetic relaxation calculations were performed for 10,000 steps following each stretch to monitor real-time stress changes and to record key mechanical property parameters. To prevent unphysical bonding phenomena, the system automatically detected and removed chemical bonds longer than 1.5 Å. The stresses of the model were quantitatively analyzed by calculating the average stress value and its standard deviation for each tensile stage.

## 3. Results and Discussion

### 3.1. Cross-Linking Analysis

#### 3.1.1. Generated Cross-Linking Model

**Figure 5:**
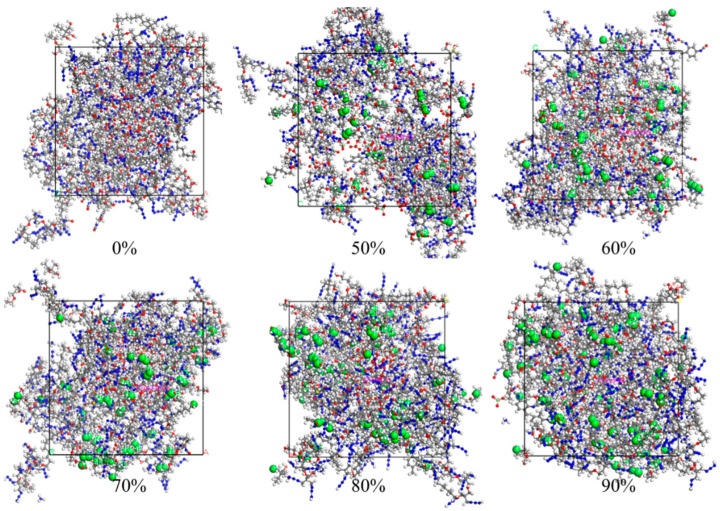
Cross-linking model diagram with different cross-linking densities. Color scheme: Gray (Carbon), Blue (Nitrogen), Red (Oxygen), Gyan (Chlorine), White (Hydrogen), Green (cross-linking atoms).

The cross-linking models of PBT, TDI, TEG, and TMP were generated through the dynamic cross-linking simulations executed via Perl script files. Figure 5 illustrates the developed cross-linking models with the cross-linking densities of 0%, 50%, 60%, 70%, 80%, and 90%. Each cross-linking system is characterized by a typical three-dimensional network configuration, with cross-linking sites visualized as green spheres. As the cross-linking density increased from 0% to 90%, the number density of cross-linking sites in the system exhibited a significant upward trend, while the spatial distribution of these sites remained uniform. This uniformity indicates that the cross-linking results from the simulations were satisfactory.

**Figure 6:**
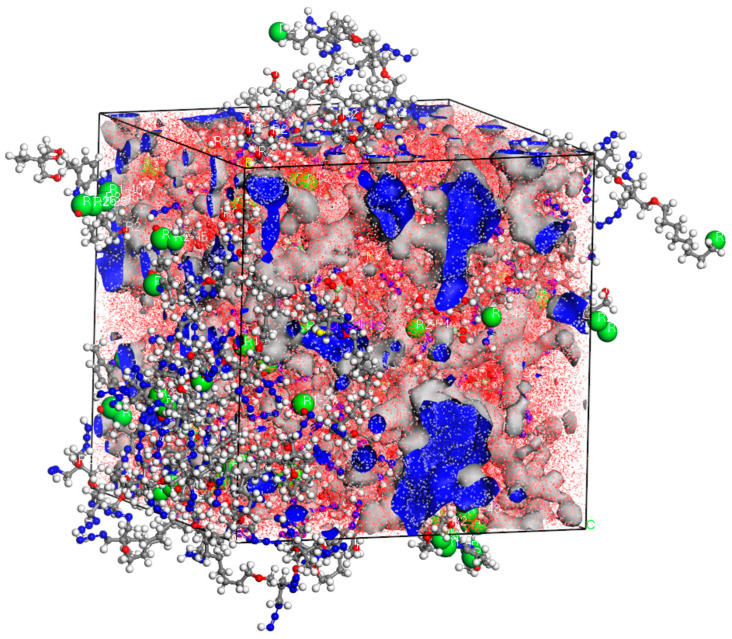
Free-volume diagram at 90% cross-linking density. Color scheme: Gray (Carbon), Blue (Nitrogen), Red (Oxygen), Gyan (Chlorine), White (Hydrogen), Green (cross-linking atoms).

Table 1 summarizes the density and free-volume characteristics of the post-cross-linking models compared to the un-cross-linked model at a temperature of 300 K. It was observed that as the cross-linking density increased, the system’s density also increased, while the free volume decreased. This behavior can be attributed to the cross-linking reaction, in which small molecules in the system are bonded together by cross-linking bonds. The resulting enhanced intermolecular interactions lead to a more compact packing structure, thereby increasing the density. Additionally, as the cross-linking reaction progresses, the molecular interactions diminish, and the distance between molecules decreases, resulting in a reduction in free volume. Figure 6 presents a schematic diagram of the free volume of the model at a 90% cross-linking density. Similar findings have been reported in the studies by Xie [31] and Ren [32].

#### 3.1.2. Cross-Linking Process Analysis

**Figure 7:**
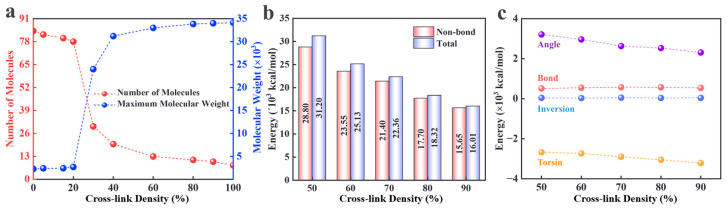
(**a**) Number of independent fragments and maximum molecular weight. Energy changes during cross-linking; (**b**) total energy and non-bonding energy; (**c**) individual terms in bonding energy.

To investigate the effect of varying cross-linking densities on the network structure, the number of monomers, independent fragments, and molecular weight of the largest molecule in the final cross-linked structure were determined for samples with different cross-linking densities. As shown in Figure 7a, the molecular weight exhibits a significant increasing trend as the cross-linking density rises from 0% to 90%. In the low-cross-linking-density range (0–20%), the number of monomers and independent fragments remains relatively stable, and the maximum molecular weight is lower. However, as the cross-linking density increases, the number of monomers and independent fragments decreases sharply, while the maximum molecular weight increases rapidly. This suggests that a higher cross-linking density promotes the polymerization of molecular chains. Upon reaching the high-cross-linking-density region (70–90%), the molecular weight continues to increase, while the number of monomers and independent fragments approaches zero. This indicates that most of the molecules have formed a more stable macromolecular network structure.

The cross-linking process is concomitant with energy state alterations. As shown in Figure 7b, as the cross-linking density increases from 50% to 90%, the total energy of the system exhibits a significant decreasing trend, closely mirroring the trajectory of the non-bonding energy. The total energy decreases by 40%, indicating that the formation of the cross-linked network leads to the densification of the molecular chain structure, which in turn weakens the non-bonding energy. The observed energy change during the cross-linking process is primarily attributed to the non-bonding energy. Additionally, the changes in bond energies should not be overlooked. As shown in Figure 7c, the evolution of the individual bonding energy components demonstrates notable variability. The gradual decrease in the bond energies is directly related to the optimization of the bond lengths resulting from the reorganization of the C-N/C-O bonds during the cross-linking process. The change in the torsional energy reflects the limiting effect of the cross-linked network on the conformational flexibility of the molecular chain; the formation of rigid structures reduces the frequency of bond angle deviations from their equilibrium positions. In contrast, the bond angle energy and reversal energy did not exhibit significant changes (fluctuations within the range of <± 2.1%) across the entire cross-linking density range (50–90%), suggesting that these energy components are less sensitive to the structural composition of the network.

#### 3.1.3. Radial Distribution Function Analysis

**Figure 8:**
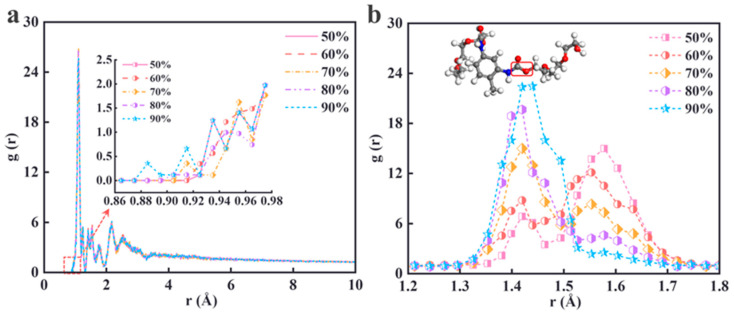
RDF diagrams at different cross-linking densities. (**a**) All-atom RDF; (**b**) C-O atomic structure and RDF at the cross-linking site.

The radial distribution function (RDF) is a key statistical tool for characterizing the microstructure of matter. It is defined as the ratio of the probability density of particles found at a distance r from a reference particle to the average density of the system, denoted as *g*(*r*). By analyzing the peak position, peak shape, and integral area of the RDF curve, structural features such as the bonding distance between atoms/molecules, coordination number, and local ordering can be quantitatively revealed, providing essential information for analyzing the static structure of materials. Figure 8a shows the RDFs of all the atoms for the five different cross-linking density systems. The RDFs of the five cross-linking density models exhibit similar characteristics. In the short range (<1 Å), the RDF values for all the models are zero due to the exclusion volume effect. In the middle and long-range regions (>5 Å), the RDFs gradually converge to a constant value (*g*(*r*) ≈ 1), indicating that, at greater distances, the distribution of particles tends to be uniform, and the local atomic density ratio to the average system density becomes constant. The earlier appearance of the RDF peaks and the denser peaks in the models with higher cross-linking densities suggest that the molecular chains are more tightly connected with more cross-linking points, resulting in stronger short-range interactions between atoms. This leads to the earlier appearance of RDF spikes and higher peaks at smaller values of *r* (atomic spacing). This phenomenon indicates that the molecular chains in high-cross-linking-density materials exhibit greater order at close proximity, with a more uniform and compact molecular arrangement.

To further investigate the cross-linking-site atoms, Figure 8b presents the RDF plot for the carbon (C) atoms in the [-NCO] group and oxygen (O) atoms in the [-OH] group. The peak at 1.4 Å corresponds to covalent bonding between the carbon atoms and other non-hydrogen atoms, particularly the carbon–oxygen or carbon–nitrogen interactions within the PBT molecular chain. As the density of the cross-linker increases, the number of cross-linking points on the PBT molecular chain rises, leading to a gradual increase in the peak in this region with higher cross-linking densities. The peak at 1.6 Å primarily corresponds to covalent bonding between nitrogen atoms in the TDI molecule. At higher cross-linking densities (90%), the saturation of the cross-linking reaction of TDI results in a decrease in the peak height at this distance.

#### 3.1.4. Diffusion Coefficient Analysis

**Figure 9:**
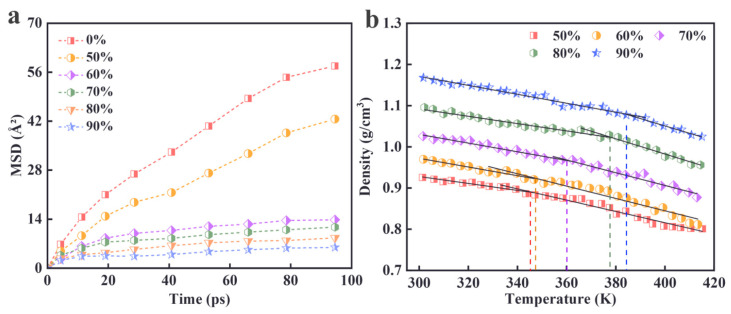
(**a**) Effect of cross-linking density on MSD; (**b**) temperature density curves at different cross-linking densities.

The mean square displacement (MSD) curve visually represents the motility and diffusion properties of the molecular chain segments by tracking the displacement evolution of particles over time. A steeper slope of the curve indicates the better motility of the polymer molecular chain segments [33,34]. The MSD curves for the system at each cross-linking density are shown in Figure 9a. As depicted, the mobility of the polymer molecular chain segments decreases progressively with increasing cross-linking densities. At lower cross-linking densities, the motility of the molecular chains is higher; however, as the cross-linking density increases, the freedom of movement of the molecular chains becomes restricted, resulting in decreased motility. This is reflected in the gradual reduction in the slope of the MSD curve. In addition, as the density of cross-linking increases, the change in the MSD changes from a rapid decrease to a slow decrease. When the density of cross-linking exceeds 60%, the slope of the MSD is very small, indicating that the molecules diffuse very slowly. These observations suggest that the cross-linking process enhances the structural rigidity of the material by increasing the chemical bonding between the molecular chains, thereby reducing their motility.

#### 3.1.5. Glass Transition Temperature Analysis

The glass transition temperature (*Tg*) directly reflects the thermodynamic properties of a material and its mechanical behavior changes, and it determines its temperature range for use, processing performance, and dynamic response characteristics. It is a key indicator for assessing the rigidity and flexibility of a material. To further investigate the effect of cross-linking density on the cross-linked network structure of PBT, this study examines the density–temperature relationship curves for five different cross-linking densities and analyzes their corresponding *Tg*.

The specific method of the *Tg* is as follows: A temperature simulation is performed on the cross-linked system from 420 K to 300 K, with a temperature interval of 10 K, cooling rate of 10 K/100 ps, and pressure of 1.01 × 10^5^ Pa. Two 50 ps dynamics simulations are performed at each temperature (100 ps in total). The first 50 ps dynamics process eliminates the local internal stress of the model while allowing the density of the system to reach equilibrium. The second 50 ps dynamics process is used to analyze the density characteristics, and other information such as temperature, volume, density, etc., during the cooling process is recorded for the analysis of the *Tg*. The first kinetic process aimed to eliminate local internal stresses in the model and ensure that the system’s density reached equilibrium. The second kinetic process was employed to analyze the density characteristics, with other parameters such as temperature, volume, and density recorded during the cooling process. These data were then used to calculate the *Tg* with each kinetic simulation lasting 50 ps.

Figure 9b presents the bilinear fitting curve, which consists of two straight lines. The intersection point of the two lines corresponds to the *Tg* value. From the figure, it can be observed that the glass transition temperature of the PBT cross-linked lattice system lies within the range of 340 K to 390 K. Furthermore, as the cross-linking density increases, the *Tg* also increases. The cross-linked model exhibits a *Tg* increase of 40–60 °C [35] compared to that of pure PBT, indicating that the rise in the cross-linking density elevates the glass transition temperature of the structure. This can be attributed to the fact that cross-linking restricts the movement of polymer molecules, requiring more energy to transition the polymer chains from the glassy to the rubbery state, which results in a higher *Tg* as the density of cross-linking increases.

### 3.2. Analysis of Tensile Results

#### 3.2.1. Effect of Cross-Link Density on Stress–Strain

**Figure 10:**
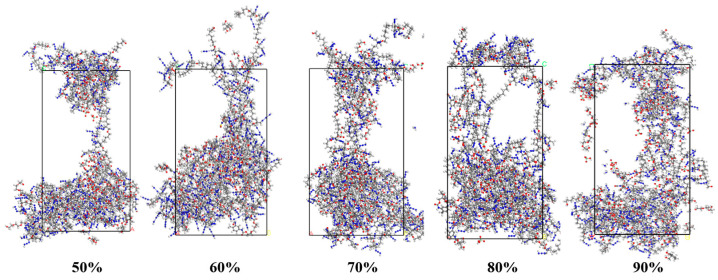
Tensile test of different cross-linking densities. Color scheme: Gray (Carbon), Blue (Nitrogen), Red (Oxygen), Gyan (Chlorine), White (Hydrogen).

Figure 10 shows the molecular structural evolution of the PBT matrices with cross-link densities of 50%, 60%, 70%, 80%, and 90% under the same strain. As the strain gradually increases from 50% to 90%, the arrangement of the molecular chains gradually changes from the initial tight entanglement to a grid arrangement. The cross-linked network gradually begins to stretch from being loose and entangled. The strain is more evenly distributed. The observations reveal that at a cross-link density of 50%, the molecular chains remain relatively flexible, exhibiting some elongation and bending. This flexibility indicates that, at lower cross-linking densities, there are fewer chemical bonds between the molecular chains, allowing for greater freedom in stretching and deformation.

At a cross-link density of 60%, the stretching of the molecular chains decreases, and the shapes of some chains begin to exhibit increased rigidity. Local cross-linking points start to emerge, although some stretching still occurs. The motility of the chain segments is more restricted compared to that of the 50% cross-link density system. At a 70% cross-link density, the molecular chains become significantly more rigid, with an increased number of cross-linking points resulting in reduced stretching. The diminished bending and deformation of the chain segments suggest that the cross-linked network is beginning to dominate, making the molecular chains less deformable than in the lower-cross-link-density system. At an 80% cross-link density, the molecular chains exhibit even greater rigidity, and the cross-linked network becomes more tightly structured. The elongation and deformation of the molecular chains are severely restricted, with a significant increase in the cross-linking points in localized areas. Consequently, the movement of the molecular chains is almost completely inhibited, leading to the stabilization of the overall structure. At a 90% cross-link density, the molecular chains are nearly fully cured, displaying extremely high rigidity. The density of the cross-linking points prevents any noticeable elongation or deformation of the molecular chains, resulting in a stable structure for the entire system. At this stage, the molecular chains form a highly organized cross-linked network, with almost no capacity for free movement.

**Figure 11:**
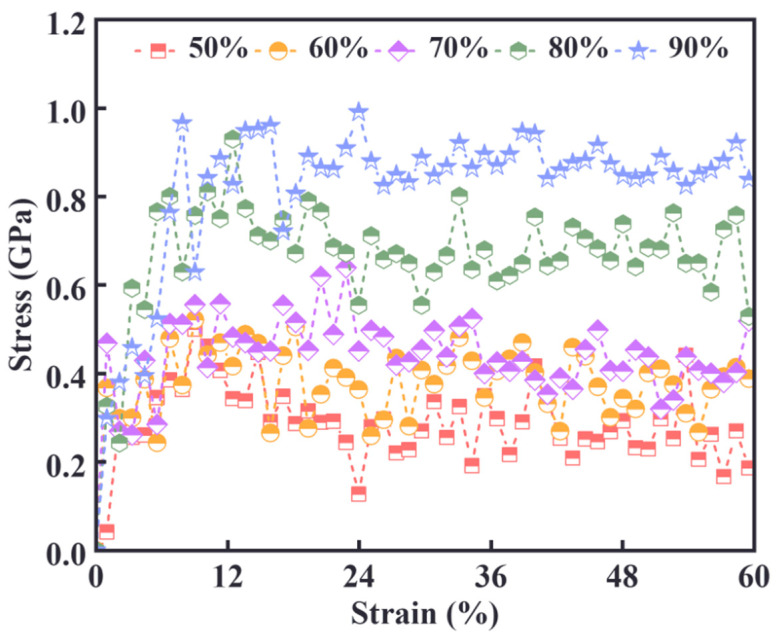
Stress–displacement curve for different densities of cross-linking.

The stress–displacement curve obtained from uniaxial stretching serves as an effective means of correlating the molecular structure with the mechanical properties of the system. As shown in Figure 11, in the initial part of the curve, stress increases rapidly with strain, particularly in the low-strain region. During this phase, the molecular chains in the cross-linked network begin to stretch under the applied external forces.

At lower cross-link densities (e.g., 50%), the stress rises more gradually, whereas at higher cross-link densities (e.g., 90%), the stress increases more sharply. Following this initial rise, stress fluctuations begin to emerge. These fluctuations are primarily attributed to the partial breakage, slippage, or localized plastic deformation of the chain segments, which are often associated with the nonlinear deformation behavior of the material. In lower-cross-link-density systems, the network structure tends to be looser, resulting in more pronounced stress fluctuations.

As the strain continues to increase, the stress begins to stabilize and levels off, particularly in systems with high cross-link densities (e.g., 90%), where the stress gradually stabilizes and fluctuations decrease. This behavior is due to the increased compactness and elasticity of the network structure, which allows the stress to stabilize at a more constant value. In contrast, lower-cross-link-density samples continue to exhibit significant fluctuations at higher strains, indicating that the material structure is not sufficiently stable. This suggests that the fracture and reorganization processes may persist in the material, preventing stress from stabilizing.

At higher cross-link densities, the connections between molecular chains become more tightly bonded, resulting in a more rigid network structure. However, once the applied strain exceeds a certain threshold, the stretching and slippage of the molecular chains are restricted by the network structure. Consequently, the cross-linking points cannot be stretched further, leading to a plateau in the stress response. The stress–strain curve of high-cross-link-density materials typically exhibits a steady state after reaching a certain strain, indicating that the cross-linked network has reached its maximum deformation capacity. The plateau phase in the stress–strain curve arises from stress redistribution through microcrack formation and localized chain breakage, which impede further stress growth and result in a stabilized stress level.

#### 3.2.2. Effect of Strain Rate on Cross-Linked Meshes

**Figure 12:**
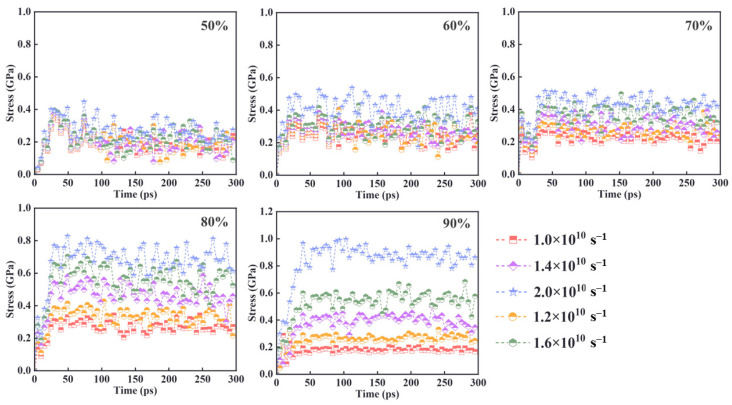
Stress–time curves at different stretching rates.

Figure 12 illustrates the stress transformation over time for different crosslink density models at varying tensile rates. In the two low crosslink density models (50% and 60%), the material structure is relatively looser, and the interactions between molecular chains are weaker. Due to insufficient cross-linking, the molecular chains can slip and relax more freely during stretching, which leads to larger stress fluctuations. This is particularly evident in the 50% crosslink density model, where the lower crosslinking density results in greater susceptibility to deformation and reduced elasticity.

At 90% crosslink density, the material structure is much tighter, with the molecular chains almost completely bound by cross-links. This leaves little to no free chain segments to slide or relax during stretching, resulting in minimal stress fluctuations and a continuous increase in stress, which leads to higher strength. Comparing the five graphs reveals that higher tensile rates cause a rapid increase in stress, with a greater growth rate. Specifically, the 90% crosslink density system shows a 42.1% higher stress growth rate compared to the 50% crosslink density system, which shows only an 18.7% increase. This indicates that higher tensile rates force the material to undergo greater deformation in a shorter period, thereby generating more stress. This suggests that tensile rate significantly influences the material’s strength, with higher rates rapidly increasing the stress response.

Additionally, for the 50% crosslink density system, stress shows a decreasing trend after reaching the peak value. This decline indicates that the material has a looser structure and weaker chain segment connections, making it less capable of withstanding stress. When strain is applied, the material quickly enters the nonlinear deformation region, where fracture and damage occur, leading to a drop in stress. In contrast, models with crosslink densities of 60% or higher exhibit a greater ability to withstand loads. The higher crosslink density materials are more structurally stable, maintaining a better stress state even under larger strains. This demonstrates that enhancing the crosslinked structure reduces the likelihood of localized damage and fracture, allowing high crosslink density systems to endure greater stress.

## 4. Conclusions

This study analyzed the cross-linking process and mechanical properties of PBT-based solid propellants using molecular dynamics simulations. The primary focus was on the cross-linked lattice composed of PBT, TDI, TMP, and TEG, examining the effects of the radial distribution function (RDF), mean square displacement (MSD), and energy changes during the cross-linking process on the material structure. Additionally, the models were subjected to various stretching rates to assess their mechanical behavior. The main conclusions are as follows:

(1) Reduced Motility and Increased Rigidity: Increasing the cross-linking density leads to a gradual reduction in the motility of the molecular chain segments, resulting in enhanced rigidity. This is reflected in a denser system with a reduced free volume and stronger interactions between the molecular chains, making the structure more compact.
(2) Increased Glass Transition Temperature: The glass transition temperature (*Tg*) increases with the cross-linking density.
(3) Energy Reduction: The cross-linking process reduces the system’s energy by approximately 40%, with the non-bonding energy being the primary contributor to this decrease.
(4) Improved Mechanical Properties: As the cross-linking density increases, the mechanical properties of the PBT-based solid propellant improve significantly. Higher cross-linking densities result in greater structural rigidity and tensile strength. Materials with higher cross-linking densities exhibit greater resistance to deformation and higher rigidity under the same strain, with significantly improved stress responses at high tensile rates.
(5) Enhanced Tensile Resistance: At higher cross-linking densities (e.g., 90%), the tensile resistance of the material is notably enhanced, characterized by higher stress values and reduced stress fluctuations compared to those of the lower-density models. In contrast, low-cross-linking-density materials show greater stress fluctuations, while high-density materials maintain a more stable stress response.
(6) Stress Stabilization During Stretching: During stretching, cross-linked mesh materials exhibit a plateau in the stress curve after an initial increase, where stress stabilizes due to the inability of the cross-linking points to stretch further. This stabilization results from microcracks and fractures within the network, leading to stress redistribution. Increasing the cross-linking density reduces the likelihood of such microcracks and fractures, thereby enabling the material to withstand greater stress.

In summary, this study provides a comprehensive analysis of the effects of cross-link densities on the mechanical properties and microstructural characteristics of azide polyether propellants, specifically focusing on PBT-based systems. Future research could benefit from exploring the long-term stability and aging effects of these cross-linked networks under various environmental conditions, as well as investigating the impact of different cross-linking agents and their concentrations on mechanical performance. Additionally, a more in-depth analysis of the dynamic behavior of molecular chains during deformation could provide further insights into the complex mechanisms governing the material’s response to stress.

## Figures and Tables

**Table 1:** Density and free volume of systems with different cross-linking densities.

Cross-Link Density (%)	Density (g/cm^3^)	Free Volume (Å^3^)	FFV (%)
0	1.00	12,437.14	19.43
50	1.05	11,234.74	17.55
60	1.11	10,424.41	16.29
70	1.13	9863.15	15.41
80	1.14	9641.23	15.06
90	1.18	8537.21	13.33

## Data Availability

Data will be made available on request.
